# Comment on Detection of Mycotoxins in Patients with Chronic Fatigue Syndrome *Toxins* 2013, *5*, 605–617

**DOI:** 10.3390/toxins8110324

**Published:** 2016-11-07

**Authors:** Mark J. Mendell

**Affiliations:** California Department of Public Health, Richmond, CA 94804, USA; Mark.Mendell@cdph.ca.gov

The paper by Brewer et al. (2013) has a key methodologic flaw [1]. The control group selected was inappropriate, resulting in an invalid comparison and findings.

The essence of a case-control study is to compare a case group having a disease with a group from the same general source population that did not develop the disease, but had the same opportunity to develop the disease and be included in the case group. When the case and control groups are compared, differences in exposure may suggest possible causes of the disease, or factors associated with causes.

In [1], diagnosis of chronic fatigue syndrome (CFS) was apparently the sole criterion of selection for the cases, which seems appropriate [1]. After inclusion, over 90% of cases were found to have biomarkers of exposure to specific fungal toxins of interest, which were suspected of involvement in causing the disease. After inclusion, most also reported a history of exposure to water damaged buildings (WDB), where these toxin exposures are presumed to have occurred. The reported WDB exposure, in over 90% of the cases, was not related to their original selection as a case group. The controls, on the other hand, were defined as “[h]ealthy control patients with no known toxic mold exposures in water-damaged buildings.” Thus controls were free of CFS and also *without reported history of exposure to WDB environments*, the presumed source of the toxin exposures. 

An appropriate control group would have consisted of individuals without diagnosed CFS, chosen as much as possible from a population who might have ended up in the case group if they had developed CFS. To exclude from the controls those *without opportunity for the exposure of interest* is completely inappropriate. This control selection strategy, aside from making the results invalid, suggests the authors may not have understood the essential purpose and requirements of a case-control comparison. Normally, a case-control study of the disease and exposures of interest in this study would be conducted by comparing a group of people with CFS diagnosed by specific criteria, and a group without diagnosed CFS. There would be *no* consideration, in the selection of either cases or controls, of what exposures the subjects thought they had been exposed to. That would involve a very subjective and imprecise way to select subjects, might have little to do with actual exposures, and most importantly, would likely introduce bias into the analysis.

It is not evident that other types of control groups would be preferable. For instance, controls who had CFS but were not knowingly exposed to WDB would give you limited useful information. The reported exposures would have no demonstrable association with disease since all the subjects would have the disease, but the results would show, among people with diagnosed CFS, whether thinking you had prior WDB exposure was associated with specific mycotoxin exposures. Alternatively, investigating whether reporting prior WDB exposure was associated with higher biomarkers of fungal mycotoxins, but in groups selected without respect to disease and not biased by this association, would be an interesting but different study.

It is important to point out that the problems with the study are related not to the selection of cases, but only to the selection of controls. Proper selection of cases but inappropriate selection of controls can make a case-control comparison invalid. I would hope that in their response, Dr. Brewer et al. deal clearly and directly with the issue of the control group selection, and provide their explicit opinion on the issue of whether the stated use in the study of both non-CFD status and non-WDB history to select controls was correct. (Apparently the only epidemiologist involved in the original paper, Dr. Madison, has died, so she cannot respond, and the remaining authors may not fully understand the criticisms or be able to respond to this question.) Also, despite the statement in the original comment by Dr. Osterman (2016) that the case-control comparison was “rigged,” that is not an issue that can be or needs to be resolved [2]. The important issue is the invalid control selection, regardless of whether due to intention or error. 

While a claim may be made that the article by Brewer et al. (2013) was only a reported case series and not intended to be an epidemiologic case-control study, this is not a credible claim [1]. The researchers studied a diseased group, and the “results were compared to healthy control subjects previously reported by the same testing laboratory.” The comparison group was defined as “[h]ealthy control patients with no known toxic mold exposures in water-damaged buildings.” Their urine specimens “were used to develop reference data for the control group used in this study.” Mycotoxins “in the urine of patients and controls were statistically analyzed to determine if a difference existed between the two groups.” So even if the authors, including the epidemiologist, somehow did not realize their study would be read as an epidemiologic case-control comparison, this will be the universal interpretation of readers, and this is how the paper should be evaluated. 

I think it would be unfortunate if Brewer et al. (2013) were cited as documenting a relationship between CFS and a body burden of mycotoxins [1]. This relationship may or may not exist, but this paper has not shown evidence to support it. I would advise the journal that in the future, review of any submitted manuscript about toxins that involves an epidemiologic study should include careful epidemiologic review. 
